# Antibiotic Use and Respiratory Viral PCR Testing Among Pediatric Patients With Nosocomial Fever

**DOI:** 10.7759/cureus.37759

**Published:** 2023-04-18

**Authors:** Carlo Foppiano Palacios, Eric Lemmon, Katelyn E Donohue, Mark Sutherland, James Campbell

**Affiliations:** 1 Medicine, Cooper University Hospital, Camden, USA; 2 Internal Medicine and Pediatrics, University of Maryland Medical Center, Baltimore, USA; 3 Emergency Medicine and Critical Care, University of Maryland School of Medicine, Baltimore, USA; 4 Infectious and Tropical Pediatrics, University of Maryland Medical Center, Baltimore, USA

**Keywords:** antibiotic administration, viral multiplex pcr, inpatient fever, pediatrics, viral infection

## Abstract

Objective

Pediatric patients admitted to the hospital often develop fevers during their inpatient stay, and many children are empirically started on antibiotics. The utility of respiratory viral panel (RVP) polymerase chain reaction (PCR) testing in the evaluation of nosocomial fevers in admitted patients is unclear. We sought to evaluate whether RVP testing is associated with the use of antibiotics among inpatient pediatric patients.

Patients and methods

We conducted a retrospective chart review of children admitted from November 2015 to June 2018. We included all patients who developed fever 48 hours or more after admission to the hospital and who were not already receiving treatment for a presumed infection (on antibiotics).

Results

Among 671 patients, there were 833 inpatient febrile episodes. The mean age of children was 6.3 years old, and 57.1% were boys. Out of 99 RVP samples analyzed, 22 were positive (22.2%). Antibiotics were started in 27.8% while 33.5% of patients were already on antibiotics. On multivariate logistic regression, having an RVP sent was significantly associated with increased initiation of antibiotics (aOR 95% CI 1.18-14.18, p=0.03). Furthermore, those with a positive RVP had a shorter course of antibiotics compared to those with a negative RVP (mean 6.8 vs 11.3 days, p=0.019).

Conclusions

Children with positive RVP had decreased antibiotic exposure compared to those with negative RVP results. RVP testing may be used to promote antibiotic stewardship among hospitalized children.

## Introduction

Pediatric patients admitted to the hospital are often tested with multiplex real-time polymerase chain reaction (PCR) respiratory viral panels (RVPs), as many cases of community-acquired pneumonia in children are due to viral pathogens [1,2]. Prior studies evaluating the impact of multiplex PCR assays for identifying respiratory viruses on antibiotic usage in children have varied depending on the clinical population, setting, and timeline of their hospitalization [3]. In the emergency department setting, testing for respiratory viruses has not been associated with a difference in the initiation of antibiotics [4]. On initial hospital admission and within two days of hospitalization, past studies found mixed results: some suggesting an association with RVP testing and antimicrobial usage while others have not [2,5-8]. However, little is known about the effect of RVP results on the treatment of nosocomial fever during a hospital stay after admission [8-10].

Inpatient pediatric patients may develop fevers during their inpatient stay [11]. Most pediatric fevers are due to self-limited viral infections [12]. Inpatient febrile episodes may be related to the child’s reason for admission, with infection being a frequent reason for hospitalization in pediatric patients [13]. Due to concern for underlying serious infection, patients are often evaluated with bacterial cultures, viral testing, and imaging [12,14]. Many children are empirically started on antibiotics, particularly those with increased severity of illness [15]. Antibiotic stewardship in children is critically important, as antimicrobial use may lead to colonization with multidrug-resistant organisms, increased length of stay and healthcare costs, and adverse effects from antimicrobial exposure, including drug toxicity and *Clostridioides difficile* colitis [16].

There is a lack of data on the utility of RVPs in the evaluation of febrile episodes among inpatient children. We sought to assess whether children who had RVP sent were more likely to be started on antibiotics and whether RVP results affected the duration of antibiotics among those started on antibiotics.

## Materials and methods

This retrospective chart review was conducted on pediatric patients (birth to 21 years old) admitted to the University of Maryland Medical Center between November 2015 and June 2018. The hospital is a 757-bed tertiary care center with all pediatric specialties, including hematology-oncology, cardiology, cardiac surgery, and neonatal and pediatric intensive care. A febrile episode was defined as a temperature greater than 38.0° Celsius. All pediatric patients who had a fever 48 hours or greater after their presentation to the hospital and were not already receiving treatment for a presumed infection (on antibiotics) were included to capture nosocomial febrile episodes. All nosocomial fever events from 48 hours after admission through hospital discharge were included in the analysis. For patients with multiple fever events during their admission, we considered new febrile episodes that occurred 14 days after the initial febrile episode. There was no specific protocol for the diagnosis and treatment of nosocomial fever in pediatric patients before or at the time of this study.

Data collected from the electronic medical record through chart review included demographic information, admission diagnosis, past medical history (PMH), and diagnostics at the time of the febrile episode (including white blood cell count, C-reactive protein, blood cultures, cerebrospinal fluid cultures, urine cultures, stool cultures, wound cultures, RVP results, other viral testing, inpatient coded diagnosis of fever, and antibiotics prescribed. During the period of this study, the microbiology lab used the xTAG® RVP for use with the Luminex 200 System, manufactured by Luminex Molecular Diagnostics, Inc., Toronto, Ontario. The turnaround time for results (after receiving samples in the laboratory) was 24 hours. The xTAG® RVP is a qualitative nucleic acid multiplex test that detected 12 viral targets: influenza A subtype H1, influenza A subtype H3, influenza B, respiratory syncytial virus subtype A, respiratory syncytial virus subtype B, parainfluenza 1, parainfluenza 2, parainfluenza 3, human metapneumovirus, rhinovirus, and adenovirus.

Descriptive statistics were used to describe patient characteristics, the laboratory studies sent for the evaluation of febrile episodes, and the management factors of febrile episodes. Demographic characteristics and inpatient unit during febrile episodes were compared to whether RVP was sent using the chi-squared test. Welch's analysis of variance (ANOVA) was used to compare if RVP was sent and RVP results with days of antibiotic therapy. A multivariate regression analysis on the initiation of antibiotics was performed, only factors that demonstrated an association with a p-value <0.10 on bivariate analysis were included: temperature, unit, PMH, C-reactive protein (CRP), and blood and sputum culture results. A predictive model was used for the multivariate logistic regression analysis: the factors with the highest p-values were removed until only factors with a p-value < 0.05 remained. The remaining factors were assessed for interaction. All data analysis was conducted using Microsoft Excel® (Microsoft Corporation, Redmond. WA) and R version 4.0.2® (R Core Team (2021). R: A language and environment for statistical ## computing. R Foundation for Statistical Computing, Vienna, Austria). This study was approved by the University of Maryland, Baltimore Institutional Review Board.

## Results

Six hundred seventy-one patients met the criteria of at least one nosocomial fever. Children were predominately African American (n=336, 50.0%), boys (n=383, 57.1%), with a mean age of 6.3 years (Table 1). The most common underlying conditions of patients included chronic lung disease (n=108, 16.1%), congenital heart disease (n=83, 12.4%), cancer (n=63, 9.4%), and prematurity (n=41, 6.1%). The mean hospital length was 38.0 days ± 46.8 days. The most common reason for admission in patients with nosocomial fever was for a planned procedure or a surgical diagnosis (n= 135, 21.6%), birth (n= 145, 21.6%), or suspected or known infection (n= 87, 12.9%). As several patients had one or more febrile episodes at least seven days apart, a total of 833 nosocomial febrile episodes were included.

**Table 1:** Patient characteristics (n=671)

	N	%
Age – mean (SD)	6.3 (7.9)	7.9
Sex (n=669)		
Female	287 (42.9)	42.9
Male	383 (57.1)	57.1
Race		
Black or African American	336	50.0
White	212	31.5
Asian	17	2.5
Other	107	15.9
Hispanic or Latino	65	9.7
Length of stay – mean (SD)	38.0 (46.8)	
Past medical history		
None	260	38.7
Chronic lung disease	108	16.1
Congenital heart disease	83	12.4
Malignancy	63	9.4
Prematurity	41	6.1
Genetic disease	35	5.2
Sickle cell	27	4.0
Asthma	15	2.2
Other	61	9.1
Admission reason		
Infection	87	12.9
Surgical diagnosis or procedure	135	20.1
Newborn	145	21.6
Respiratory disease (non-infectious)	51	7.6
Cancer-related condition	58	8.6
Gastrointestinal condition	42	6.2
Cardiac disease	41	6.1
Sickle cell-related condition	25	3.7
Neurological disease	28	4.2
Other	60	8.9

**Figure 1:**
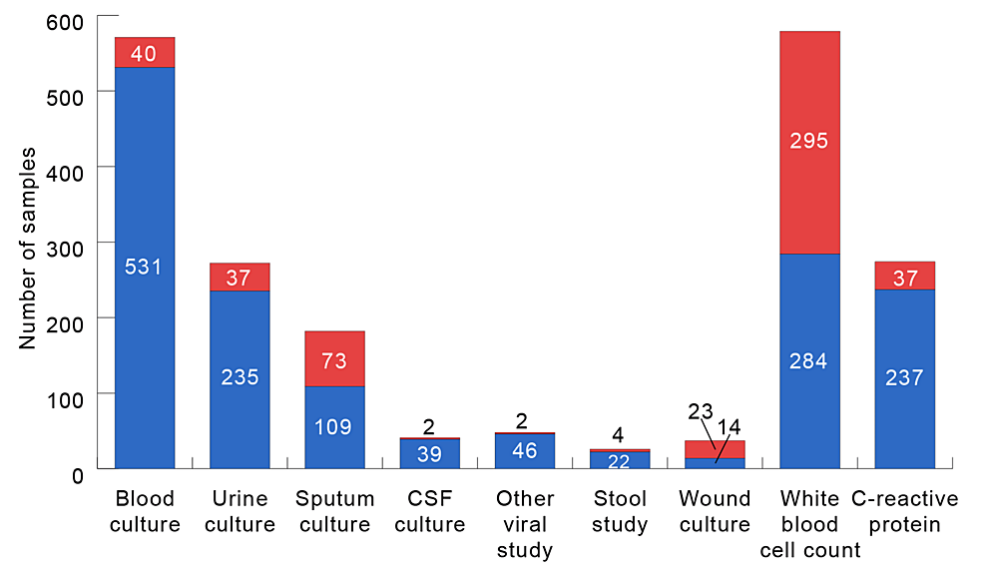
Laboratory studies sent for the evaluation of a febrile episode Red: positive result or culture growth noted. For white blood cell count, red represents a level > 11 (109/L). For C-reactive protein, red represents a level > 10 (mg/L). Blue: negative result or sterile culture noted. For white blood cell count, red represents a level ≤ 11 (109/L). For C-reactive protein, red represents a level ≤ 10 (mg/L).

The majority of the 833 nosocomial fevers occurred while in the neonatal intensive care unit (NICU) (n=286, 34.3%), followed by the pediatric intensive care unit (PICU) (n=263, 31.6%) (Table 2). Due to the absence of an institutional testing protocol for inpatient fevers at the time of the study, labs were sent varied for each febrile episode (Figure 1). An RVP was sent within 24 hours of the fever in 11.9% of febrile episodes (n=99/833). Patients were more likely to have an RVP sent if they were aged seven months to two years (11.3% vs 27.3%, p<0.001) and had a past medical history (25.3% vs 35.8%, p=0.04), a history of congenital heart disease (13.4% vs 21.2%, p=0.05), or blood (7.0 vs 14.3%, p=0.07) or urine (10.4% vs 25.5%, p=0.02) cultures sent. Of the ninety-nine RVPs sent, 22.2% (n=22/99) were positive for a viral infection. At the time of nosocomial fever, 41.8% (n=348/833) of patients were started on antibiotics, and 52.2% (n=485/833) were not. After a febrile episode, 58.0% (n=202/348) of the children remained on antibiotics for two days or less. The mean length of antibiotic therapy was 8.2 ± 7.0 days.

**Table 2:** Management of a febrile episode RVP: respiratory viral panel

	N	%
Inpatient unit during febrile episode		
Neonatal intensive care unit	286	34.6
Pediatric intensive care unit	263	31.6
Pediatric floor	185	22.2
Other units	99	
RVP sent (n=99)		
Positive RVP	22	22.2
Negative RVP	77	77.8
Antibiotics started at the time of a febrile episode		
Antibiotics not started	485	52.2
Antibiotics started	348	41.8
Days of antibiotics (n=348)		
2 days or less	202	58.0
3-7 days	125	35.9
8-14 days	0	0.0
Greater than 14 days	21	6.1

On bivariate analysis, patients who had nosocomial fever with an RVP sent were more likely to be started on antibiotics (n=78/99) than those without an RVP sent (n=270/734, p<0.001). On multivariate logistic regression, the initiation of antibiotics was associated with having RVP sent (aOR 3.68, 95% CI 1.18-14.18, p=0.036) and having growth on sputum cultures (aOR 10.64, 95% CI 3.31-48.04, p<0.001). The duration of antibiotic use was not statistically different between nosocomial fever with an RVP sent compared to nosocomial fever without an RVP sent (p=0.46, Table 3). However, when evaluating only those who had an RVP sent (n=99), patients who had a positive RVP (6.8 ± 4.9 days) had a significantly shorter course of antibiotics than those with a negative RVP (11.3 ± 11.1 days, p=0.019).

**Table 3:** RVP and antibiotic use RVP: respiratory viral panel

	RVP not sent (N=734)	RVP sent (N=99)	p-value
Antibiotics started at the time of a febrile episode			p<0.001
Antibiotics not started	464 (63.2)	21 (21.2)	
Antibiotics started	270 (36.8)	78 (78.8)	
Days of antibiotics – mean (SD)	8.4 (7.0)	7.7 (6.8)	p=0.46

The viruses isolated by RVP included rhino/enterovirus (n=12/22, 54.5%), respiratory syncytial virus (n=7/22, 31.8%), adenovirus (N=3/22,13.6%), parainfluenza virus (N=2, 9.1%), influenza B virus (N=1, 4.5%), and human metapneumovirus (N=1, 4.5%). Four patients tested positive for two viruses: one tested positive for RSV and rhino/enterovirus, another tested positive for rhino/enterovirus and influenza B, and two tested positive for both rhino/enterovirus and adenovirus. The difference in RVP test results across seasons was not statistically significant.

Among all nosocomial fevers, no definite etiology was identified in 50.4% (n=420/833) of febrile episodes. For patients who had a nosocomial fever with an identified cause, the most common diagnoses were bacterial infections (n=155/833, 18.6%) or post-procedure fever (n=96/833, 11.5%).

## Discussion

To our knowledge, this is the first study that evaluates the use of RVP testing among hospitalized inpatient children who develop a fever greater than 48 hours into their hospital course. Sending an RVP assay was strongly associated with the initiation of antibiotics among hospitalized children who developed a fever during their hospital course. Additionally, patients with a positive RVP result had a decreased length of antibiotic use after a febrile episode compared to those with a negative RVP.

There are limited data published on the practices surrounding the workup and treatment of nosocomial fever in pediatric patients [14]. We found that patients who had an RVP sent were more likely to be started on antibiotics than patients who did not have an RVP sent. Children who had an RVP sent may have been perceived as being more vulnerable to serious bacterial infections and therefore started on antibiotics. Prior data showed that children with complex chronic conditions are more likely to be diagnosed with and treated for pneumonia [17]. Furthermore, the children who had an RVP sent were more likely to have blood or urine cultures sent, likely representing concern for serious bacterial infection.

Among patients with an RVP sent, those with a positive RVP had decreased exposure to antibiotics compared to those with a negative RVP. Our data support prior studies that have demonstrated an association between RVP testing and antimicrobial use among children on admission to the hospital [18-21]. Testing inpatient children who develop a fever and have viral upper respiratory symptoms can help identify patients with a low risk of serious bacterial infections [22]. If a respiratory virus is isolated, inpatient children may not need systemic antibiotic therapy [23]. RVP testing may be used to promote antimicrobial stewardship among hospitalized children who develop a fever due to viral infections [10,24].

Our study had several limitations. First, data were collected from a retrospective review of inpatient medical records at a single academic medical center. Our data set included a small number of positive RVPs (n=22), limiting the ability to analyze antibiotic use in this group and compare the baseline parameters and any differences in demographic characteristics between those with positive and negative RVP. We included all patients with fever 48 hours into hospital admission in our analysis. It is possible that some nosocomial febrile episodes were related to the original reasons for admission. Thus, we may have included children who had an infection on hospitalization and are persistently febrile 48 hours later. The data were collected before the onset of the COVID-19 pandemic, and the epidemiology of respiratory viruses among admitted children who develop a fever may be significantly different now. At the time of the study, the turnaround time on RVP results was 24 hours, which may have affected the duration of time patients were on antibiotics compared to receiving a more immediate result. Additionally, this study was not powered to perform subgroup analyses.

Future work should evaluate the utility of inpatient RVP testing for high-risk subgroups of pediatric patients, such as young infants, including those in the NICU, and patients with underlying medical conditions such as malignancy, congenital heart disease, or chronic lung disease. Our study did not assess the impact of RVP on imaging acquisition, days in isolation, or its impact on patient mortality. As rapid multiplex PCR testing becomes increasingly available, future studies will need to compare how these new assays impact costs of care and clinical outcomes. Finally, a prospective randomized controlled trial is needed to evaluate whether inpatient RVP testing affects patient outcomes, including mortality.

## Conclusions

Children with nosocomial fever who had an RVP sent were more likely to be started on antibiotics. Patients with a positive RVP had decreased antibiotic exposure compared to those with negative RVP results. RVP testing may be used to promote antibiotic stewardship among hospitalized children.
